# The Stressful Life of the Urban Consumers: The Case of Dhaka City Residents

**DOI:** 10.3389/fpsyg.2021.747414

**Published:** 2021-11-17

**Authors:** Muhammad Rehan Masoom

**Affiliations:** School of Business and Economics, United International University, Dhaka, Bangladesh

**Keywords:** perceived stress, materialistic value-orientation, locus of control, religiosity, structural equation model, urban consumers, Bangladesh

## Abstract

The present research examines the metropolitan mental life of consumers of Dhaka, which is one of the most densely populated and least livable cities in the world. Though mental life encompasses a range of factors, the study considered the dynamic interplays of the most pertinent ones, such as perceived stress, the sense of control, materialistic values, and religiosity. These variables were measured and quantified by commonly used measurement tools; a recursive structural equation model was constructed to unearth the causal connections among those variables. By using a 57-item questionnaire, the study surveyed 1,068 shoppers living in 10 different zones of the city. The estimated covariance by the multivariate structural equation model indicates that perceived stress is significantly associated with the sense of control, while religiosity and materialistic value-orientation were negatively associated. However, there are no significant relationships between religiosity and sense of control, and materialism and sense of control. Perceived stress and religiosity are found to be positively associated. The estimated independent sample *t*-tests showed that while no significant difference is found in sense of control by gender, women were more religious, less materialistic, but perceive their lives as more stressful than the men. The findings help to interpret both the cognitive and affective responses of the consumers of urban residents.

## Introduction

The last decade has been a period of multi-level consumer revolution because of the presence of social media that form public relationships at various levels (Quesenberry, 2020). While sustained economic growth leads to a surge in discretionary expenditure on globally branded consumer items, the income distribution has become more and more unequal at the same time (Davis, 2005). The purchase process in urban retail chains affects the psychic state or the mental life of the consumers (Halkin, 2018). Consumers adjust their buying habits to cope with the stress that comes with the transitions in urban life (Lee et al., 2001). While from the social and cultural perspective, cities always generate some unpleasant feelings such as stress, discomfort, and animosity (Mubi Brighenti and Pavoni, 2019), retail crowding (i.e., consumer response to human density) has also been an intriguing area of study by marketing science (Eroglu et al., 2005). Particularly, Neuromarketing, the emerging discipline in marketing science, emphasizes studying the cognitive and affective responses of consumers (Lee et al., 2007). Consumer dispositions affect the linkages between stress-related appraisals, consumption feelings, and the coping process (Duhachek and Iacobucci, 2005). Stress can exacerbate detrimental tendencies, such as materialism and compulsive purchase (Burroughs and Rindfleisch, 2002). Nonetheless, the mental life, particularly the perceived stress of the consumers, has received little attention in studying consumer behavior (Moschis, 2007). The present research examines the metropolitan mental life of consumers of Dhaka by focusing on the dynamic interplays of the most pertinent psychosocial factors, such as perceived stress, sense of control, materialistic values, and religiosity.

The mental life of urban consumers reflects a set of organized attitudes and sentiments that may differ from one person to another in countless obvious ways, but their metropolitan “mental life” requires them to embrace and maintain the objective existence against the overwhelming forces of the city (Tole, 1993). Like most of the megacities, Dhaka is not merely “a physical mechanism and an artificial construction but the vital processes of the people who compose it; it is a product of nature, and particularly of human nature” (Park et al., 1984, p. 01). The residents, like the residents of other megacities, require maintaining the autonomy and uniqueness of their human nature in the face of constant and overwhelming transformation of the urban social structure (Mridha and Moore, 2011). Perhaps, the rapid transformation of Dhaka encourages the urbanites to liberate themselves from the shackles of all historical bonds, be it religious, political, economic, or moral (Bertuzzo, 2009). They may live in “a mosaic of little social worlds which touch but do not interpenetrate” (Park et al., 1984, p. 26). Perhaps, the major problem may not be the absence of social solidarity or the loss of communal values, but the sense of powerlessness (Seeman, 1971, p. 140). That powerlessness may lead the Dhaka residents to adopt a collaborative approach, with the fellow residents and with the divine (Pargament, 1997). Religious beliefs and commitment help them in self-preservation and retrain them from committing any destructive social conduct (Pargament et al., 1998). All these, with the unifying motives in the narrower sense of survival, form the inseparable whole of the metropolitan mental life of the consumers. Therefore, there should be a shared internal means by which they deal with their immediate external social environment. Their unique escalated awareness and a predominance of intelligence lead them to respond with “their heads instead of their hearts” (Simmel and Wolff, 1950, p. 01). However, it remains a question of what essentials elements of their mental life could be, and what is the nature of the dynamic interplays of those elements.

We are inclined to believe that the urban environment shapes the cognitive characteristics of the consumers; their sense of control, which is either internal (high) or external (low), manifests their assessment of personal control over outcomes (Fiori et al., 2006). Because of the ever-increasing complexity of the urban environment, the urban consumers require confronting “the latent, and often unintended, consequences of one’s actions” (Geyer, 2001, p. 388). Since the complexities of urban lives are appearing to be perceived as arbitrary and unpredictable, the urbanites determine their social priorities to preserve their sense of selfness and powerlessness (Ross et al., 2001). This sense of powerlessness denotes their view toward their capability to affect and regulate outcomes of their life experiences (Foulk et al., 2020). However, the confidence of handling the social problems of urban lives, controlling irritations of social lives, and the feelings that social life is heading in a favorable direction enhances self-efficacy and reduces the sense of helplessness (Cohen et al., 1993, 1994). Here, religion becomes a socially determined means that helps them not to be terrified of their imponderable urban social conditions (Hancock and Srinivas, 2008).

Religion in the urban environment is more of a perspective and less of the strict codes of conduct; religion seems to be “the heart of the heartless” (Marx, 1844) urban life, and the means to express the mild aversion, reciprocal strangeness, and repulsion by the residents. Religious organizations, such as mosques, offer the opportunity to interact in a way that the urbanites feel that they have positive social relations with everyone all around (Durkheim, 1915). Particularly, in religious ceremonies, they interact with those who have been the neighbors for years and yet are often not even identified by sight in everyday interaction (Durkheim, 1915). However, any metropolis is based upon an integration of several distinctive phenomena, and the market economy is the most pronounced one (Simmel and Wolff, 1950). “A city is a state – of mind, of taste, of opportunity. A city is a marketplace – where ideas are traded, opinions clash and eternal conflict may produce eternal truths” (Caen, 1967, p. 08). The urban environment favors the practice of reciprocity, where material items have a social meaning and value beyond their functional use (McFarlane, 2011). Materials become the objective measurable achievement of self-esteem whereby the metropolitan citizen reckons with whom he must have social ties (Reeves et al., 2012). Since the multiplicity and concentration of economic transactions emphasize the means of exchange and the value it generates, money becomes the shared concern (Sirgy, 1999). With a very distinct sensation to the materialistic urban lives, the psychic behavior always reacts to almost any perception of the materialistic values. Therefore, the current research hypothesized that the urban environment of Dhaka offers a greater opportunity to form materialism and yet religion remains the essential aspect of social lives. The interplays between materialistic values and religiosity were hypothesized to be critically related to perceived stress and the sense of control.

## Background and Hypotheses

Perceived stress is the perceptions or ideas of an individual about the amount of stress they are experiencing at any particular time or during a specified period (Phillips, 2013, p. 1453). Perceived stress includes sentiments of the uncontrollability and imprevisibility of life, how often one faces annoying troubles, how many changes one makes in his or her life, and how confident he or she is to address the hurdles or problems faced (Asberg and Renk, 2014). It is not the measurement of types or frequencies of a person when it comes to strained events, rather how a person feels about the overall stress of life and the ability to cope with these stresses (Phillips, 2013). Because of factors including personality, coping resources, and support, people perceive the effect or severity of comparable life events differently (Asberg and Renk, 2014). Perceived stress thus reflects the interplay between an individual and their environment that they view as threatening or exhausting their resources (Lazarus and Folkman, 1987). Perceived stress is frequently quantified using a questionnaire such as the Perceived Stress Scale (PSS-10) to determine the frequency of such feelings (Cohen et al., 1993). While perceived stress was once thought to be a one-dimensional assessment, the academic community now considers it to be two-dimensional (Taylor, 2015); the negatively phrased items implying perceived helplessness, and the positively phrased things implying perceived self-efficacy (Hewitt et al., 1992). There is some evidence that the degree of psychological and biological responses to stress is influenced by the impression of control over stressful circumstances (Maes et al., 1998). Some research looked at the impact of stressor control perception on both subjective and physiologic stress responses (Bollini et al., 2004). Sense of control is the belief that one has control over their own life rather than external influences (Ross et al., 2001). The sense of control can be understood as internal, i.e., the belief that one can control their life, or external, that is the view that life is controlled by external elements that the person have no impact on or that chance or destiny rules his or her life (Rotter, 1966). The internal locus of control tends to praise or criticize oneself; the external locus of control tends to praise or blame external causes (Anderson, 1976). Based on these key self-evaluation dimensions, the following hypothesis was made:

H1: Consumers with high perceived stress have a low sense of control.

The perceived power to control life events and outcomes are shaped by religious beliefs and actions (Fiori et al., 2006). Religiosity is a defining factor of social lives, wherein the preferences of people when it comes to apparel, foods, social gatherings, and decision-making often depend on their religious beliefs and religious commitment (Sethi and Seligman, 1993). It is “the degree to which beliefs in specific religious values and ideals are held and practiced by an individual” (Delener, 1990). People assess the significance of their religion in a variety of ways that range from inclusive (beliefs and spirituality) to exclusive (rituals and practices) (Büssing, 2017). Religious people are less inclined to regard difficulties as threats as their religious beliefs help them cope with undesirable life events (Ellison, 1991). Their faith in divine power or God strengthen the function of creating a sense of security; they believe that God will ensure that “all goes well,” or at the very least will go well in the future; this reduces stress and contributes to general well-being (Ellison and Burdette, 2012). The conviction that one is loved and treasured by God is linked to self-worth (Crocker and Nuer, 2003). Therefore, we hypothesize the following:

H2: Consumers with high religiosity have low perceived stress.

It is important to note that there are two competing theories to explain the effect of religion on the sense of control. The relinquished control thesis argues that religious belief will reduce the sense of control of an individual because heavenly creatures govern life occurrences and results (Jackson and Coursey, 1988). To believe in God as an active agent, one must give up personal or internal control (Jackson and Coursey, 1988). Relying on supernatural intervention can diminish mastery by distracting from problem-solving attempts (Ferraro and Kelley-Moore, 2001). Invoking God to cope with stress may reduce self-efficacy (Pargament, 1997). Conversely, for the personal empowerment thesis, religion and mastery are linked positively. Belief in an omnipotent supernatural being may increase human power and lessen uncertainty (Pargament, 1997). The fact that God is on their side does not lessen their sense of efficacy or mastery, wherein those who lack power may not need to rely on other kinds of self-efficacy (Jackson and Coursey, 1988). Along with religious beliefs, private religious practice can enhance a sense of stability and coherence. Praying is essential to creating a relationship with the divine (Demir, 2019). The relationship with God provides hope, control, and general peace (Ellison et al., 2012). Prayer can help people feel more valued and in control, as they are appreciated and helped by a divine force (Ellison, 1991). Personal prayer influences to reduce depressive symptoms and anxiety, as well as enhance self-esteem (Maltby et al., 1999). Furthermore, reading religious writings may help explain why religiousness is linked to higher levels of life satisfaction, in which readers may feel connected to the characters in the literature, especially if they share difficulties (Ellison, 1991). Individuals may also feel “less alone” in battling their challenges if they relate them to a biblical figure (Levin, 2008). Religious role-taking might provide ideas about how to behave and think to overcome challenges (Ellison et al., 2012). Therefore, the following prediction can be made:

H3: There is a significant association between religiosity and the sense of control.

The market economy, which is intrinsically connected to the mentality of the urbanites, forms materialistic value-orientation. Materialism is a state of mind that expresses an obsession with acquiring and spending (Rassuli and Hollander, 1986). It is the forming experiences of those who regard their belongings as an extension of themselves (Belk, 1985). To the materialist, possession is a goal of life, and they develop “Acquisition Centrality,” where “possessions assume a central place in life of a person and are believed to provide the greatest sources of satisfaction and dissatisfaction” (Belk, 1985; p. 291). Possessions have social significance not only through their utilitarian function in supporting daily life, but also as symbols of identity, personality, and self-expression (Dittmar and Pepper, 1994). Financial prosperity, admirable assets, the perfect impression made by consumer goods, and a high standing measured by the size of the wallet of an individual are all culturally sanctioned aspirations of materialistic values (Kasser et al., 2004). When materialism becomes a dominating normative value, people prioritize material comfort over core requirements such as social life quality or a sense of belongingness (Richins, 1994b). Profit, power, efficiency, and competitiveness have emerged as the primary motivators in the daily lives of people (Belk, 1985). The beliefs of an individual, their dedication to, and reverence of a divine are affected by materialistic value-orientation (Gallagher and Tierney, 2013). Materialism and Religiosity are two of the most incompatible yet dominating components of normative value-systems that are constantly in confrontation with each other (Masoom and Sarker, 2017a). It is found that religious individuals are less materialistic (Burroughs and Rindfleisch, 2002) because if religious people adopted materialistic principles, they would begin to perceive their lives as less meaningful (Dorn, 2014). Tangible objects are perceived as barriers to spiritual transcendence (Kavanaugh, 1997; Smith et al., 2003). However, religion remains a powerful factor in changing economic times and states of conflict, and it may have a major effect on materialistic value-orientation (Rakrachakarn et al., 2015). Therefore, the following hypothesis was proposed:

H4: Highly religious consumers have low materialistic value-orientation.

The quantity and nature of the possessions are one of the most noticeable ways in which people appear affluent (Dittmar, 1992). However, the materialists did not necessarily feel that they can regulate the outcomes of their life events; they may suffer from lower levels of well-being, spend money on a range of non-required purchases, have high debt, and low financial savings (Watson, 2003), problematic interpersonal relationships (Richins and Dawson, 1992), and high levels of substance use (Vansteenkiste et al., 2007). Materialists try to make up for their inability to achieve their desired self-esteem by emphasizing material accomplishment that eventually prevents them from concentrating on more intrinsically satisfactory goals. Arguably, the pursuit of material possessions is a self-defeating cycle (Richins, 1994a); when an individual buys something that has some materialistic values, it satisfies his or her desire. However, a new desire is spawned as he or she acclimatizes to it, and a more prestigious and thus potentially costlier purchase may now be needed to fulfill the desire. The desire will then be revived, and the self-defeating process repeats until the individual can no longer fulfill it or assumes a great deal of debt to do so. In either case, the leverage of the person over fulfilling the urge to benefit is gradually becoming weaker. A dependence on possessions to build self-worth solidifies the vulnerability of individuals to factors such as social acceptance (Kashdan and Breen, 2007); they often become reluctant to be in touch with negatively assessed thoughts and emotions, and thereby strive to escape circumstances. Besides, materialism was inversely linked to competence and autonomy, where these two constructs are linked to feelings of power (Eisenberger et al., 1999). Materialism was positively linked to negative feelings but was not linked to positive feelings (Kashdan and Breen, 2007). Hence, we can hypothesize the following:

H5: Highly materialistic consumers have high perceived stress.

H6: Highly materialistic consumers have a low sense of control.

The hypotheses mentioned above are tested in a recursive model in which the effects flow in one direction with no feedback loops, indicating that the effects are sometimes also the causes. The recursive model is estimated in structural equation modeling (SEM) by examining variances and covariances to find interrelationships among the four proposed latent variables. Besides, men and women differ by their perceived stress, sense of control, religiousness, and materialistic value-orientation. Several studies reported that compared with men, women experience higher levels of chronic and daily pressures (Culhane et al., 2001; Gentry et al., 2007; Anbumalar et al., 2017). It was found that compared with men, women view their interpersonal relations and life events were more controlled by external factors (Sherman et al., 1997). Regardless of the religious background, it was found that women were more religious than men (Schnabel, 2015; Masoom, 2020). Likewise, studies indicate that men are more likely than women to believe that having material possessions boosts their pleasure and allows them to express themselves more freely (Browne and Kaldenberg, 1997; Segal and Podoshen, 2013). Hence, we can hypothesize the following:

H7: Compared with men, women are more religious, less materialistic, have less sense of control, and perceive a higher level of stress.

The proposed hypotheses are being tested in the context of Dhaka city, which is the city that is one of the most densely populated (23,234 inhabitants per km^2^ in a gross surface of 300 km^2^) metropolises in the world. Approximately 75% of the residents are literate, about 23% are unemployed, and approximately 90% are Muslims and their life expectancy is nearly 71 years^1^. Due to its historical significance, Dhaka can be considered a classic example of a metropolis. Once, it was the capital of the Mughal Empire, an administrative province of eastern India, and famous for its cotton industries. By the early 18th century, Dhaka had lost its status as the provincial capital, and by the second half of the 18th century, the city lost its position as a significant center of administration, commerce, and development (Ahmed and Islam, 2021). Dhaka became the capital of eastern Pakistan after the partition of India-Pakistan in 1947. After the liberation war of Bangladesh, the shape of Dhaka approached that of a conurbation in the late 1970s in the sense that its external growth began to connect with outlying suburbs (Bertuzzo, 2009). The expansion of the city from the 1980s onward meant filling up the adjacent empty spaces to meet the needs. The iconic parliament complex, palaces, mosques, temples, roads, bridges, gates, gardens, aqueducts, tanks, and modern markets are the pronounced features of the present Dhaka city. Here, the life of the ordinary people, commonly termed as middle-class, is non-eventful and invariably the same within the limited opportunities offered by the city (Lefebvre et al., 1996). Most of the residents are Muslims, and Islam is a faith that is presumed to have strong effects on Muslim culture (Kavoossi, 2000). The enforcement of religious rules and regulations in the legal structures of many Islamic countries has a clear and profound impact on the lifestyles and consumption habits of Muslims (Cyril De Run et al., 2010). For example, a study in Bangladesh showed that increasing materialistic values have a decreasing impact on the level of religiosity (Masoom and Sarker, 2017b).

## Materials and Methods

### Research Design and Sampling

To unveil that latent mechanism of the mental life of the urban consumers, the study quantified the four variables, namely, perceived stress, sense of control, religiosity, and materialistic values, in a way that facilitated the statistical procedure. No new item was generated, and the Likert-scale format was used, as proposed by the relevant established scales used in the research. IBM SPSS Statistics (Version 23.0. Armonk, NY: IBM Corp) and IBM SPSS Amos graphics (Version 24. Chicago: IBM Corp) were used to perform the statistical analysis.

The study used *superpopulation* model as an alternate way of random sampling (Isaki and Fuller, 1982). By *superpopulation* model, the population could be attributed as a finite number and the sample could be attributed as unbiased predictors to meet the requirement to make any statistical inferences. Out of a total of 50 sub-offices for the postal service in Dhaka city, 10 were selected by lottery. From the selected postal zones, the prominent shopping malls were enlisted, and one was selected by lottery. To formulate the *superpopulation*, the surveyors counted the number of people visiting the selected malls to make a rough estimation of the number of shoppers. After the population was counted, each k^th^ shopper had been considered as the respondent if consent was given. At least 100 respondents were considered from the selected shopping malls of each postal zone. The value of k was determined by the total number of shoppers of a particular shopping mall divided by 100. The target sample size was no less than 384 men and 384 women since the size would be adequate by 95% confidence level (CL) and 5% CI. The targeted total sample size was at least 1,000 respondents as that would be adequate by 99% CL and 5% CI. At the time of the survey, the surveyors informed the participants that their participation is voluntary, and no personal information will be disclosed at any point in time. It took about 20 weeks for 10 surveyors to complete the survey by using pencils and printed questionnaires.

### Instruments and Procedure

There were three demographic information-related questions (gender, age, and education), and 57-items included in the structured questionnaire corresponding to four segments to elicit the response from the target population. It would be prudent to include variables like monthly income or employment in the questionnaire. However, being mall intercept, it cannot be ensured that all participants had a sample job and a fixed income, hence, including only the basic demographic variables could reduce non-response error (Bush and Hair, 1985). While selecting the scales and the items, the internal reliability and cross-cultural validity reported in the previous studies were taken into consideration. The research used the PSS-10 (Cohen et al., 1993) to address the level of stress suffered by urban consumers. Perceived self-efficacy and perceived helplessness were treated as two-factor formative constructs because of their validity and reliability in cross-cultural contexts (Siqueira Reis et al., 2010), including the sample from Dhaka city (Masoom and Hoque, 2018). The scale is one of the most reliable (Cronbach’s alpha > 0.7) measurement tools to address perceived stress (Andreou et al., 2011; Maroufizadeh et al., 2014).

To measure sense of control, which is sometimes termed as the locus of control in social sciences, the study considered the Locus of Control of Behavior Scale (LCB) (Craig et al., 1994) because it was reported not to be affected by social desirability problems, and also found to yield strong internal reliability (Cronbach’s alpha > 0.8) (Hooley, 1998; Sagone and De Caroli, 2014). Recent research argues that two dimensions, namely perceived powerlessness and perceived control, increased the psychometric properties for the LCB scale (Bright et al., 2013). The study of Richins and Dawson (1992) most successfully measured the degree of “Materialistic Value-Orientation” by three constructs, which were acquisition centrality, material-driven success, and materialistic happiness with the 18-item scale (Richins and Dawson, 1992). Since previous research (cf. Masoom and Sarker, 2017b) indicated that the items corresponding to constructs of the materialism scale did not load in the developing economy context like the original scale prescribed, the present research changed the association of the items of the relevant constructs when required. The study has selected 12 items (five items for religious beliefs and seven items for religious commitment) to measure religiosity from the “Religiousness Scale” (Sethi and Seligman, 1993). It was one of the most comprehensive tools to investigate the level of religiosity of people with different faiths, and was found to be a valid measure for the Bangladesh Sample (Masoom, 2020).

Religiosity and materialism were measured on a 7-point Likert scale, and sense of control was measure on a 6-point Likert scale, where 1 indicated responses such as Strongly Disagree, Not at all influential, or Strongly Disbelieve, whereas the maximum value (6 or 7) indicates Strongly Agree, Extremely Influential, or Strongly Believe. The higher values of these three constructs indicate the lower sense of control, higher religiosity, and higher materialism. Perceived stress was measured on a 5-point Likert scale, where 0 indicated never and 4 indicated very often. We measured the construct validity of the four variables by confirmatory factor analysis (CFA) before estimating the dynamic interplays among the four variables. All four variables were considered as two-dimensional second-order formative constructs in the multivariate recursive model. In addition, we measured the means and *SD* of the items, construct-wise grand means, and calculated the independent sample *t*-test to explore the factor-wise and item-wise differences by gender.

## Results

### Participants

A total of 1,068 Dhaka city consumers participated in the survey, approximately 36.2% of the surveyed were women, and about 80% of the participants were less than 30 years old. Less than 5% of the surveyed were above 50 years old. Approximately 30% of the respondents were undergrad students. Please consider Table 1 for the demographic detail of the respondents.

**TABLE 1:** Demographics of the sample (*n* = 1068).

Factor	Category	Frequency	Percent
Gender	Male	681	63.8
	Female	387	36.2
	Total	1068	100.0
Age	Less than 20	69	6.5
	20–29	802	75.1
	30–39	104	9.7
	40–49	48	4.5
	50–59	34	3.2
	Above 60	11	1.0
	Total	1068	100.0
Level of Education	Higher Secondary	406	38.0
	Undergrad	321	30.1
	Graduate	216	20.2
	Post-grad	125	11.7
	Total	1068	100.0

### Psychometric Properties

A two-factor solution (perceived self-efficacy and perceived helplessness) to address PSS-10 was found valid. The validity of the scale as unidimensional measurement [CFA = 0.83; root mean square error of approximation (RMSEA) = 0.06] was less that of two-dimensional measurement (CFA = 0.94; RMSEA = 0.04). Likewise, the internal consistency of the scale as unidimensional (Cronbach’s alpha = 0.59), did not reach the acceptance level. While the reliability of perceived self-efficacy (Cronbach’s alpha = 0.40) was poor, whereas the reliability of perceived helplessness (Cronbach’s alpha = 0.66) was satisfactory. Perceived Helplessness could explain 48% of the total variation, and perceived self-efficacy could explain 56% of the variation. Hence, perceived stress was treated as a two-factor formative construct in the recursive model. The most effective item to measure helplessness was (S1) “becoming upset because of something happened unexpectedly” (β = 0.62) and to measure efficacy was (S4) “confidence of handling personal problems” (β = 0.52).

About seven items of the LCB scales were loaded poorly (<0.3) in the structural equation model, hence, those items were deleted. These items were (L2) emphasizing the matter of chance, (L3) considering luck as the determinants of future, (L10) believing that people are victims of circumstance, (L12) indicating some biological dispositions such as tightness in muscles, or (L14) irregular and fast breathing are beyond their control. Furthermore, some positively worded items such as (L13) believing that a person can really be the master of his/her fate, and (L15) indicating that why problems vary so much from one occasion to the next, yielded poor regression co-efficient. The comparative-fit index (CFI) (0.93), and RMSEA (0.03) showed that two factors (powerlessness and control) with a total of 11 items could be treated as valid. Powerlessness could explain 54% of the total variation, and control could explain 57% of the variation. Items such as (L9) attributing the outside actions and events controlling life (β = 0.53) and (L16) confidence to deal successfully with future problems (β = 0.56), were mostly influencing to measure the sense of control. The estimated indices [CFI = 0.93; goodness of fit index (GFI) = 0.98; RMSEA = 0.04] showed the validity of the construct.

Religiosity was addressed by adopting common items from the scale (Sethi and Seligman, 1993). It was a two-factor construct, namely religious beliefs (five items) and religious commitment (seven items). The importance of religious beliefs in life (RQ2) (β = 0.62) and the influence of religion on apparel wearing (RQ8) (β = 0.75) were the two influencing items that corresponded to the constructs measuring religiosity. The primary indices [i.e., CFI, GFI, and adjusted GFI (AGFI)] reached the thresholds (>0.9). The internal consistency for religious beliefs (Cronbach’s alpha = 0.64) and religious commitment (Cronbach’s alpha = 0.78) was satisfactory.

The CFI of Richins and Dawson (1992) proposed measurement for materialistic value-orientation was poor (CFI = 0.55), and the standardized regression co-efficient of five items were negligible (<30% of variation explained). Hence, a new two-factor measurement tool was constructed by exploratory factor analysis. Internal consistency of the two factors, namely, acquisition centrality (seven-item factor; Cronbach’s alpha = 0.70) and acquisition simplicity (6-item factor; Cronbach’s alpha = 0.67) was satisfactory. While the CFI (0.87) was very close to the threshold, the other primary indices (i.e., GFI and AGFI) reached the thresholds (>0.9) for materialism. The most influencing item to measure acquisition centrality was (M2) “Some of the most important achievements in life include acquiring material possessions” (β = 0.57), and to measure Acquisition simplicity was (M8) trying “to keep life simple, as far as possessions are concerned” (β = 0.69).

Overall, all four variables were two-dimensional formative constructs, while the CFI for materialism was close to the threshold, all other three variables yielded acceptable values. The normal chi-square (CMIN/DF) values of these four constructs were ranging from above 2 but not exceeding 5.5. RMSEA for all four variables was good (<0.06), while the standardized root means square residual (SRMR) of all the constructs did not exceed 0.05. Therefore, all four constructs were valid measures to explore the dynamic interplays among the variables of interest. For more results, refer to Table 2.

**TABLE 2:** Validity and reliability of the measurements.

Variable(s) and its validity	Associated constructs, corresponding items, and their reliability	β
**Perceived stress** CMIN/DF = 2.66 CFI = 0.94 GFI = 0.98 AGFI = 0.97 RMSEA = 0.04 (*p* = 0.96) SRMR = 0.04	**Helplessness (AVE = 0.26; CR = 0.66; Cronbach’s alpha = 0.66)**	0.48
	S1. how often have you been upset because of something that happened unexpectedly?	0.62
	S2. how often have you felt that you were unable to control the important things in your life?	0.57
	S3. how often have you felt nervous and “stressed”?	0.47
	S6. how often have you found that you could not cope with all the things that you had to do?	0.31
	S9. how often have you been angered because of things that were outside of your control?	0.46
	S10. how often have you felt difficulties were piling up so high that you could not overcome them?	0.54
	Efficacy^*^ (AVE = 0.15; CR = 0.41; Cronbach’s alpha = 0.40)	0.56
	S4. how often have you felt confident about your ability to handle your personal problems?	0.52
	S5. how often have you felt that things were going your way?	0.37
	S7. how often have you been able to control irritations in your life?	0.31
	S8. how often have you felt that you were on top of things?	0.34

**Locus of control** CMIN/DF = 2.48 CFI = 0.93 GFI = 0.98 AGFI = 0.97 RMSEA = 0.04 (*p* = 0.98) SRMR = 0.03	**Powerlessness (AVE = 0.22; CR = 0.56; Cronbach’s alpha = 0.57)**	0.54
	L4. I can control my problem(s) only if I have outside support	0.30
	L6. My problem(s) will dominate me all my life	0.52
	L9. My life is controlled by outside actions and events	0.53
	L11. To continue to manage my problems I need professional help	0.52
	L17. In my case maintaining control over my problems is due mostly to luck	0.41
	Control^*^ (AVE = 0.19; CR = 0.54; Cronbach’s alpha = 0.53)	0.57
	L1. I can anticipate difficulties and take action to avoid them	0.44
	L5. When I make plans, I am almost certain that I can make them work	0.41
	L7. My mistakes and problems are my responsibility to deal with	0.43
	L8. Becoming a success is a matter of hard work; luck has little or nothing to do with it	0.33
	L16. I am confident of being able to deal successfully with future problems	0.56

**Religiosity** CMIN/DF = 5.06 CFI = 0.93 GFI = 0.96 AGFI = 0.94 RMSEA = 0.06 (*p* = 0.01) SRMR = 0.04	**Beliefs (AVE = 0.25; CR = 0.61; Cronbach’s alpha = 0.64)**	0.72
	RQ2. How important would you say religion is in your life	0.62
	RQ12. Do you believe that there a heaven?	0.58
	RQ14. Do you believe there are miracles?	0.40
	RQ15. Do you believe your suffering will be rewarded?	0.52
	RQ16. Do you believe that in the future your children will be able to lead a better life than yourself?	0.32
	**Commitment (AVE = 0.36; CR = 0.79; Cronbach’s alpha = 0.78)**	1.07
	RQ4. How often do you pray?	0.32
	RQ6. How much influence do your religious beliefs have on the important decisions of your life?	0.65
	RQ7. Would you support to marry someone of another religion?	0.47
	RQ8. How much influence do your religious beliefs have on what you wear?	0.75
	RQ9. How much influence do your religious beliefs have on what you eat and drink?	0.73
	RQ10. How much influence do your religious beliefs have whom you associate with?	0.55
	RQ11. How much influence do your religious beliefs have on what social activities you undertake?	0.62

**Materialism** CMIN/DF = 5.11 CFI = 0.87 GFI = 0.96 AGFI = 0.93 RMSEA = 0.06 (*p* = 0.01) SRMR = 0.05	**Acquisition Centrality (AVE = 0.24; CR = 0.69; Cronbach’s alpha = 0.70)**	1.00
	M1. I admire people who own expensive homes, cars, and clothes	0.46
	M2. Some of the most important achievements in life include acquiring material possessions	0.57
	M4. The things that I own say a lot about how well I’m doing in life	0.48
	M5. I like to own things that impress people	0.41
	M11. Buying things give me a lot of pleasure	0.50
	M12. I like a lot of luxury in my life	0.49
	M18. It sometimes bothers me quite a bit that I can’t afford to buy all the things I like	0.50
	Acquisition Simplicity^*^ (AVE = 0.27; CR = 0.68; Cronbach’s alpha = 0.67)	1.00
	M3. I don’t place much emphasis on the amount of material objects, people own as a sign of success	0.48
	M6. I don’t pay much attention to the material objects other people own	0.44
	M7. I usually buy only the things I need	0.56
	M8. I try to keep my life simple, as far as possessions are concerned	0.69
	M13. I put less emphasis on material things than most people I know	0.51
	M14. I have all the things I really need to enjoy life	0.39

*Items reverse coded for the model.

*All estimates are significant, *p* < 0.001.*

### Recursive Model Estimates

**FIGURE 1:**
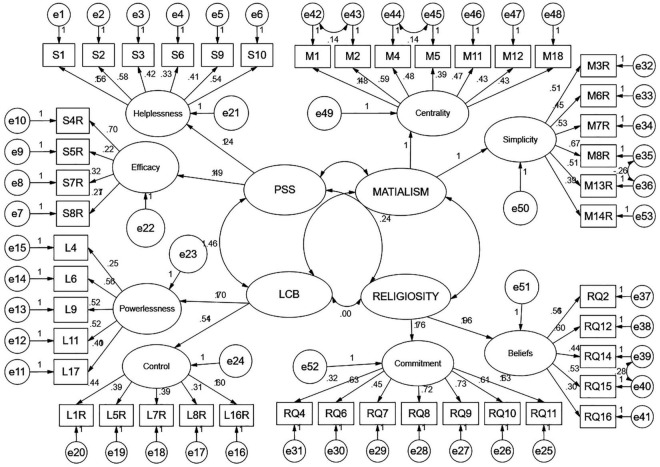
Recursive model of second-order formative constructs. Minimum iteration was achieved; chi-square = 6295.60; degrees of freedom = 2,793; and probability level = 0.001.

The estimated covariance by the recursive structural equation model showed few significant and few non-significant relationships. Perceived stress and sense of control were covarying significantly (*t* = 7.02, *p* = 0.001). Likewise, religiosity was covarying with perceived stress (*t* = 2.49, *p* = 0.013), but religiosity did not covary with the sense of control (*t* = –0.25, *p* = 0.805). While materialistic values covaried with the religiosity (*t* = –6.44, *p* = 0.001), neither perceived stress (*t* = 0.97, *p* = 0.466) nor sense of control (*t* = 0.26, *p* = 0.793) covaried with materialism. For the path-diagram of the constructed structural equation model, see Figure 1. For the degree of covariance, see Table 3.

**TABLE 3:** Estimated covariance in the recursive model.

Variable	Variable	Regression coefficient	Standard error	*t*	*p*
Perceived stress	Sense of control	0.093	0.013	7.02	0.001
Religiosity	Sense of control	−0.005	0.007	−0.25	0.805
Materialism	Sense of control	0.003	0.010	0.26	0.793
Perceived stress	Religiosity	0.034	0.014	2.49	0.013
Perceived stress	Materialism	0.007	0.007	0.97	0.338
Religiosity	Materialism	−0.112	0.017	−6.44	0.001

### Gender-Wise Mean Differences

The comparative mean values ($\bar{x}$) of the responses unveiled some basic patterns of the mental life of the consumers living in Dhaka. The estimated mean scores (2.05 < $\bar{x}$ < 2.54) of the items and constructs of perceived stress implied that the life of the Dhaka city consumers was sometimes (values around 2) stressful. While compared with men ($\bar{x}$ = 2.21), women ($\bar{x}$ = 2.31) perceived helplessness (*t* = –2.196, *df* = 1,066, *p* = 0.028). There were hardly any differences between men ($\bar{x}$ = 2.24) and women ($\bar{x}$ = 2.22) as far as self-efficacy was concerned (*t* = 0.362, *df* = 1,066, *p* = 0.718). However, there were no significant differences by gender in the degree of sense of control, and neither the two constructs such as powerlessness and control nor any of the 10 items used to address the variable showed any gender-wise significant differences. The mean values (3.41 < $\bar{x}$ < 3.92) of the negatively worded items corresponding powerlessness indicated that they were not feeling highly powerless. Conversely, the mean values (4.18 < $\bar{x}$ < 4.71) of the positively worded items corresponding control indicated that the consumers of Dhaka city perceived their life be controlled by their own decisions.

The mean values (4.05 < $\bar{x}$ < 6.22) of the items of religiosity indicated that consumers of Dhaka city were religious. Not only the religious commitment of women ($\bar{x}$ = 5) was significantly (*t* = –4.059, *df* = 1,066, *p* = 0.001) higher than the men ($\bar{x}$ = 4.71), but also the religious beliefs of women ($\bar{x}$ = 5.83) were significantly (*t* = –4.041, *df* = 1,066, *p* = 0.001) higher than the men ($\bar{x}$ = 5.57). Women ($\bar{x}$ = 4.46) were more likely than men ($\bar{x}$ = 4.05) to pray (*t* = –3.601, *df* = 1,066, *p* = 0.001), the religious beliefs of women ($\bar{x}$ = 5) influenced more than men ($\bar{x}$ = 4.53) on what to wear (*t* = –4.135, *df* = 1,066, *p* = 0.001), the religious beliefs of women ($\bar{x}$ = 5.58) influenced more than men ($\bar{x}$ = 5.30) on what to eat or drink (*t* = –2.594, *df* = 1,066, *p* = 0.01). Likewise, women ($\bar{x}$ = 6.22) gave more importance than men ($\bar{x}$ = 6.03) did on their religious beliefs (*t* = –2.309, *df* = 1,066, *p* = 0.021). Men ($\bar{x}$ = 4.9) believed less in miracles than the women ($\bar{x}$ = 5.29) did (*t* = –3.172, *df* = 1,066, *p* = 0.002), and women ($\bar{x}$ = 6.48) believed more in the existence of heaven than the men ($\bar{x}$ = 6.13) did (*t* = –4.318, *df* = 1,066, *p* = 0.001). Finally, the religious beliefs of men ($\bar{x}$ = 5.06) influenced less than women ($\bar{x}$ = 5.33) when it comes to their decision making (*t* = –2.783, *df* = 1,066, *p* = 0.005).

While there were no significant differences between men ($\bar{x}$ = 3.97) and women ($\bar{x}$ = 3.88) by acquisition simplicity (*t* = 1.7, *df* = 1,066, *p* = 0.089), men ($\bar{x}$ = 3.76) scored high on acquisition centrality than women ($\bar{x}$ = 3.59) indicating the significant gender differences (*t* = 3, *df* = 1,066, *p* = 0.003). Compared with women ($\bar{x}$ = 3.24), men ($\bar{x}$ = 3.5) admired people who own expensive homes, cars, and clothes (*t* = 2.598, *df* = 1,066, *p* = 0.010). Likewise, men ($\bar{x}$ = 3.87) liked a lot of luxury in life than the women ($\bar{x}$ = 3.65) did in their lives (*t* = 2.388, *df* = 1,066, *p* = 0.017). Women ($\bar{x}$ = 3.12) scored less than men ($\bar{x}$ = 3.43) considering the acquisition of properties as a significant accomplishment (*t* = 3.255, *df* = 1,066, *p* = 0.001). Compared with women ($\bar{x}$ = 3.63), men ($\bar{x}$ = 3.82) were more positively inclined to not emphasizing material objects as a sign of success (*t* = 2.402, *df* = 1,066, *p* = 0.018). Similarly, compared with men ($\bar{x}$ = 3.92), women ($\bar{x}$ = 3.74) were less positively inclined to not paying attention to the material objects other people own (*t* = 2.062, *df* = 1,066, *p* = 0.042). Women ($\bar{x}$ = 3.99) scored low than men ($\bar{x}$ = 4.2) on the assertion that they buy only the things that are needed (*t* = 2.137, *df* = 1,066, *p* = 0.035). For more results, see Table 4.

**TABLE 4:** Mean (*SD*) and the *t*-test statistics of men-women differences.

Item no.	Factors and items (Response points)	Mean (Standard deviation)			Test statistics		
		Total	Male	Female	t	df	*p*
**PSS**	**Helplessness** (Minimum = 0, Maximum = 4)	2.25 (0.69)	2.21 (0.68)	2.31 (0.70)	–2.21	1,066	0.028
S1	Upset by something unexpectedly	2.32 (1.14)	2.26 (1.15)	2.42 (1.13)	–2.22	1,066	0.027
S2	Unable to control life’s important things	2.25 (1.10)	2.24 (1.11)	2.26 (1.09)	–0.23	1,066	0.815
S3	Feeling nervous and stressed	2.46 (1.08)	2.43 (1.09)	2.50 (1.07)	–0.93	1,066	0.354
S6	Finding Hard time dealing with the amount of things to do	2.05 (1.08)	2.03 (1.05)	2.09 (1.12)	–0.92	1,066	0.359
S9	Angry at things that were beyond control	2.29 (1.15)	2.26 (1.15)	2.34 (1.13)	–1.06	1,066	0.291
S10	Finding Overwhelming number of difficulties piled up	2.13 (1.22)	2.06 (1.26)	2.25 (1.14)	–2.50	1,066	0.013

**PSS**	**Efficacy** (Minimum = 0, Maximum = 4)	2.23 (0.65)	2.24 (0.65)	2.22 (0.67)	0.36	1,066	0.718
S4	Feeling confident of the ability to handle personal problems	2.54 (1.09)	2.55 (1.11)	2.53 (1.04)	0.30	1,066	0.766
S5	Feeling that things were going in favor	2.09 (1.08)	2.08 (1.07)	2.12 (1.11)	–0.69	1,066	0.490
S7	Ability to control irritations in life	2.25 (1.07)	2.28 (1.06)	2.20 (1.09)	1.16	1,066	0.244
S8	Feeling of being on top of things	2.05 (1.14)	2.05 (1.15)	2.05 (1.11)	0.11	1,066	0.914

**LCB**	**Powerlessness** (Minimum = 1, Maximum = 6)	3.64 (0.88)	3.64 (0.88)	3.65 (0.88)	–0.16	1,066	0.872
L4	Problems can be controlled only by outside support	3.91 (1.42)	3.92 (1.41)	3.89 (1.43)	0.41	1,066	0.679
L6	Problems dominate the entire life	3.59 (1.51)	3.58 (1.49)	3.63 (1.53)	–0.52	1,066	0.604
L9	Life is controlled by outside actions and events	3.50 (1.45)	3.48 (1.44)	3.53 (1.47)	–0.58	1,066	0.565
L11	Professional help is needed to continue to manage problems	3.41 (1.49)	3.44 (1.50)	3.36 (1.47)	0.84	1,066	0.403
L17	Maintaining control over problems is due mostly to luck	3.83 (1.41)	3.81 (1.44)	3.87 (1.36)	–0.658	1,066	0.511

**LCB**	**Control** (Minimum = 1, Maximum = 6)	4.38 (0.77)	4.38 (0.77)	4.37 (0.78)	0.10	1,066	0.865
L1	Anticipating difficulties and taking action to avoid them	4.24 (1.35)	4.20 (1.37)	4.32 (1.30)	–1.46	1,066	0.144
L5	After plans are made, things usually turn out well	4.31 (1.23)	4.34 (1.22)	4.26 (1.24)	1.08	1,066	0.278
L7	Own mistakes and own problems are own responsibilities	4.71 (1.29)	4.69 (1.28)	4.73 (1.31)	–0.45	1,066	0.655
L8	Hard work brings success where luck has little/nothing to do	4.18 (1.39)	4.20 (1.37)	4.14 (1.43)	0.62	1,066	0.537
L16	Confident to deal with future challenges effectively	4.47 (1.24)	4.49 (1.25)	4.43 (1.23)	0.79	1,066	0.429

**REL**	**Beliefs** (Minimum = 1, Maximum = 7)	5.66 (1.03)	5.57 (1.05)	5.83 (0.96)	–4.04	1,066	0.001
RQ2	Importance of religious beliefs	6.10 (1.39)	6.03 (1.45)	6.22 (1.27)	–2.31	1,066	0.021
RQ12	Believing on the existence of heaven	6.26 (1.39)	6.13 (1.51)	6.48 (1.11)	–4.32	1,066	0.001
RQ14	Believing on the miracles	5.05 (1.94)	4.90 (1.94)	5.29 (1.92)	–3.17	1,066	0.002
RQ15	Believing on the reward for sufferings in afterlife	5.56 (1.61)	5.50 (1.65)	5.68 (1.54)	–1.79	1,066	0.073
RQ16	Believing in better tomorrow for the children	5.35 (1.63)	5.28 (1.64)	5.45 (1.62)	–1.61	1,066	0.107

**REL**	**Commitment** (Minimum = 1, Maximum = 7)	4.81 (1.16)	4.71 (1.16)	5.00 (1.13)	–4.06	1,066	0.001
RQ4	Frequency of prayer	4.20 (1.77)	4.05 (1.74)	4.46 (1.80)	–3.60	1,066	0.001
RQ6	Influence of religious beliefs on decision-making	5.16 (1.51)	5.06 (1.49)	5.33 (1.53)	–2.78	1,066	0.005
RQ7	Supporting interfaith marriage	5.22 (2.03)	5.05 (2.07)	5.54 (1.91)	–3.91	1,066	0.001
RQ8	Influence of religious beliefs on apparels	4.70 (1.78)	4.53 (1.82)	5.00 (1.67)	–4.13	1,066	0.001
RQ9	Influence of religious beliefs on what to eat and drink	5.40 (1.73)	5.30 (1.77)	5.58 (1.67)	–2.59	1,066	0.010
RQ10	Influence of religious beliefs on whom to associate with	4.37 (1.84)	4.33 (1.83)	4.43 (1.87)	–0.91	1,066	0.364
RQ11	Influence of religious beliefs on social activities	4.66 (1.61)	4.63 (1.62)	4.69 (1.59)	–0.57	1,066	0.570

**MAT**	**Acquisition Centrality** (Minimum = 1, Maximum = 7)	3.70 (0.88)	3.76 (0.84)	3.59 (0.93)	3.00	1,066	0.003
M1	Admire people who own expensive homes, cars, and clothes	3.41 (1.62)	3.50 (1.55)	3.24 (1.71)	2.60	1,066	0.010
M2	Acquisition of properties is a significant accomplishment	3.32 (1.53)	3.43 (1.51)	3.12 (1.54)	3.25	1,066	0.001
M4	Material belongings say a great deal of people’s whereabouts	3.20 (1.55)	3.23 (1.52)	3.15 (1.60)	0.77	1,066	0.440
M5	Like to own things that impress people	3.64 (1.53)	3.66 (1.54)	3.60 (1.53)	0.64	1,066	0.523
M11	Buying things give a lot of pleasure	3.99 (1.46)	3.99 (1.44)	3.99 (1.49)	0.05	1,066	0.962
M12	Like a lot of luxury in life	3.79 (1.43)	3.87 (1.41)	3.65 (1.46)	2.39	1,066	0.017
M18	Unaffordability of the liked things bothers quite a bit	4.00 (1.42)	4.05 (1.41)	3.90 (1.44)	1.59	1,066	0.112

**MAT**	**Acquisition Simplicity** (Minimum = 1, Maximum = 7)	3.94 (0.84)	3.97 (0.82)	3.88 (0.86)	1.70	1,066	0.089
M3	Not emphasizing material objects as a sign of success	3.75 (1.25)	3.82 (1.22)	3.63 (1.28)	2.40	1,066	0.018
M6	Not paying attention to the material objects other people own	3.86 (1.36)	3.92 (1.35)	3.74 (1.40)	2.06	1,066	0.042
M7	Buying only the things that needed	4.13 (1.51)	4.20 (1.48)	3.99 (1.55)	2.14	1,066	0.035
M8	Trying to keep life simple, as far as possessions are concerned	4.17 (1.35)	4.19 (1.38)	4.14 (1.32)	0.53	1,066	0.591
M13	Putting less emphasis on material things that most known people	3.87 (1.26)	3.87 (1.26)	3.87 (1.26)	–0.01	1,066	0.996
M14	I have all the things I really need to enjoy life	3.86 (1.42)	3.84 (1.41)	3.91 (1.43)	–0.82	1,066	0.413

## Discussion

The study intended to unveil some cognitive characteristics of the urban consumers by quantifying the four variables, which were perceived stress, sense of control, religiosity, and materialistic values. It was hypothesized that consumers with high perceived stress have a low sense of control (H1), while consumers with high religiosity have low perceived stress (H2), and religiosity and a sense of control have a considerable relationship (H3). It was also predicted that highly religious consumers have a low materialistic value orientation (H4), whereas consumers with high materialistic value orientation have a high degree of perceived stress (H5), and a low sense of control (H6). Finally, it was argued that compared with men, women are more religious, less materialistic, have less sense of control, and perceive a higher level of stress (H7). These hypotheses were tested in a single recursive model by formulating an SEM and by surveying 1,068 shoppers living in 10 different zones of Dhaka city, one of the most densely populated metropolises of the world. The results suggested perceived stress was significantly associated with the sense of control, while religiosity and materialistic value-orientation were negatively associated with each other. There were no significant relationships between religiosity and sense of control, as well as materialism and sense of control. Perceived stress and religiosity were found to be positively associated. While no significant difference was found in sense of control by gender, women were more religious, less materialistic but perceive their lives as more stressful than the men. Particularly, while there was no gender-wise difference in perceived self-efficacy, the perceived helplessness of women was higher than the men. The findings of the present research squared with a range of previously known bivariate associations.

The current study suggested that consumers with high perceived stress had a low sense of control, while religiosity has no significant associations with a sense of control, consumers with high perceived stress were highly religious. Similar findings were reported in previous studies. For instance, a high level of perceived stress led to a high degree of helplessness, which contributed to the association between the sense of control and perceived stress (Asberg and Renk, 2014). Failing to control the external social environment was also found to be an important contributor that could generate stress (Sadowski and Blackwell, 1985). While perceived control did not affect biological and subjective stress responses, the acute sense of powerlessness could increase stress by reducing perceived control (Bollini et al., 2004). Materialistic individuals are not less likely to encounter positive affective states than less materialistic individuals, but they are more likely to only experience stronger negative affective states. Likewise, materialistic individuals are also more likely to perceive a high degree of powerlessness, but not necessarily perceiving a low degree of control (Christopher et al., 2009). Perhaps the actual pursuit of possessions could lead to periodic benefits in feelings of perceived control and positive effect when possessions were originally acquired. Because of this, no significant association was found between materialism and perceived stress, and materialism and sense of control. It was found that highly materialistic consumers were less religious, wherein the differences in materialism were derived from differences in religious values and devotion (Wuthnow, 2002; Bryant-Davis and Wong, 2013). With economic growth leading to conspicuous consumption, the social value of religion decreases (McCleary and Barro, 2006).

The degree of sense of control, perceived stress, religiosity, and materialistic values differ by gender. First, women are made to feel powerless through informal cycles of power and powerlessness that influence the relative availability of opportunities and resources (Atkinson and Delamont, 1990). While gender affects the status of women and their access to decision-making power, it also affects the types of expectations, perceptions, and reactions to emotional displays that keep women from advancing and gaining power in their relationships (Ryan and Haslam, 2007). The emotions of women, such as compassion, kindness, and nurturing, have less interpersonal strength than the emotions of men, such as confidence and pride (Sinaceur and Tiedens, 2006; Overbeck et al., 2010). Those in positions of power can express emotions that increase their authority in professional relationships, perpetuating the cycle of gender power disparities in the workplace (cf. Gibson and Schroeder, 2002). Second, women are compelled by social relationships to be “submissive, meek, obedient, and caring,” qualities that are associated with higher degrees of religion (Mol, 1985). Religious participation is viewed as a household activity that is primarily carried out by the woman out of care for the well-being of a family (Glock et al., 1967). Women are more religious as a result of their lower participation in the workforce and more responsibility for the upbringing of their children. Moreover, women with lower labor force involvement not only have more time for religious activities but also have a personal identity, which may compensate for their lack of social influence (Luckmann, 1967). Rejecting religious ideas is risky behavior, and because women are exposed to more danger in almost every aspect of life, they are less inclined to engage in such behavior (Bromiley and Curley, 1992). Likewise, daughters are subjected to more parental monitoring in patriarchal societies, whereas sons are comparably free to engage in risky activities. Third, the self-monitoring characteristics of men make them more materialistic than women (Cass, 2001; Lim and O’Cass, 2001). While women are more interested in fashion than men, research shows that men are more interested in durable things like cars (Bloch, 1981). Men acquire instrumental and recreational items that enhance independence and activity on the spur of the moment (Dittmar et al., 1995). Women, on the other hand, purchase symbolic and self-expressive objects that represent their appearance and emotional elements (Dittmar et al., 1995). Men indicated more functional, instrumental, and activity-related reasons, whereas women gave more emotional and relationship-oriented reasons (Dittmar, 1989). Men report higher convenience and time-consciousness, whereas women report higher shopping delight, brand awareness, pricing awareness, and shopping confidence (Seock and Bailey, 2008). Therefore, men were found to have more acquisition centrality than women.

The findings of the present research contribute to the multidisciplinary approach toward interpreting social dilemmas and extend our understanding of consumer psychology. Besides its physiological and psychological effects, perceived stress influences consumer behavior in a variety of ways (Celuch and Showers, 1991). For instance, perceived stress can make consumers immobile or passive, which reduces their intention of purchasing products that previously felt as necessary (Torres and Nowson, 2007). Conversely, stress can lead to impulsive buying (O’Guinn and Faber, 1989), changes in brand preferences (Mathur et al., 2003); it can lead to the consumption of alcohol and drugs (Heatherton and Baumeister, 1991), or a high level of fatty food intake (Oliver et al., 2000), an increase in the frequency of comparison shopping behaviors (Anglin et al., 1994), or the number of unintentional purchases (Park et al., 1989). Considering that many stressful events result from external factors outside the control of the consumer, it leads to a low sense of control over the surroundings or the consequences of actions (Cutright, 2012). Loss of control has several behavioral implications, including compensatory behavior to regain control (Cutright et al., 2013). One such activity is ratifying religious commitment, which gives people a sense of power over a divine plan (Kay and Eibach, 2013). Religious beliefs, like superstitions, provide meaning to random events (Whitson and Galinsky, 2008). Thus, if stressful circumstances caused a sense of loss of control over the environment of an individual, consumers might respond by regaining control with their religious beliefs. Another type of compensatory behavior was consumers could use their financial resources to regain control in stressful situations. For instance, saving money gives one a sense of control because it ensures that money will be there when needed. Likewise, spending money on only essentials and not on non-necessities could provide a sense of control by making essential products readily available (Durante and Laran, 2016). With hoarding behavior, consumers may engage in behavior that could make them saving money to an unhealthy extreme (Klontz et al., 2012). However, when the sense of control of consumers was challenged, they could also seek comfort and control by amassing belongings (Hartl et al., 2005). These could be physical goods gained in the past or the present monetary possessions; this could lead them to the ideology of acquisition centrality (Richins, 1994b). Therefore, consumers could seek to restore control as a response to a stressful event by either religiosity or materialistic possessions.

There is no way but to agree that it is virtually impossible to pinpoint the coherent way of the metropolitan mental life of the consumers, but it is very much possible to outline the dynamic interplays among its essential aspects. The metropolitan mental lives of urban consumers can be expressed by the dynamic interplays among perceived stress, sense of control, materialistic value-orientation, and religiosity. While the research provides an account of the modes of experiences in terms of the reactions of the inner life to the external urban conditions, we should acknowledge the limitations as far as the external validity of the findings is concerned. First, the mall intercept was a less effective method of selecting the target population, although it benefited us to select the non-poor, non-disadvantaged solvent urban consumers in quick time. Particularly, the mall intercept was less useful if we were to collect the older aged respondents. Second, we were inclined to keep the response points as the established scales deliver, but a homogenous response category, be 5-point or 7-point, could have increased the internal validity of the research. Despite the limitations, we were inclined to believe that the research findings are valid as far as the general picture of the mental life of the urban consumers is concerned. What appears explicitly as dissociation in the metropolitan style of life was the dilemma faced by residents of the metropolis that centered on the self-schema they form. The research has portrayed the degree and the mixture of this style of life, the pace of its emergence and disappearance, and the forms in which the urban life was lived by the consumers in the Dhaka metropolis.

## Data Availability Statement

The original contributions presented in the study are included in the article/Supplementary Material, further inquiries can be directed to the corresponding author/s.

## Ethics Statement

The studies involving human participants were reviewed and approved by the Institute of Advanced Research (IAR), United International University, Dhaka, Bangladesh. The Ethics Committee waived the requirement of written informed consent for participation.

## Author Contributions

MM: study conception and design, data collection, analysis and interpretation of results, and draft manuscript preparation.

## Conflict of Interest

The author declares that the research was conducted in the absence of any commercial or financial relationships that could be construed as a potential conflict of interest.

## Publisher’s Note

All claims expressed in this article are solely those of the authors and do not necessarily represent those of their affiliated organizations, or those of the publisher, the editors and the reviewers. Any product that may be evaluated in this article, or claim that may be made by its manufacturer, is not guaranteed or endorsed by the publisher.

## Footnotes

1
worldpopulationreview.com
